# Response to Dr. DiCarlo‐Cohen

**DOI:** 10.1002/acm2.14046

**Published:** 2023-05-22

**Authors:** Jacqueline P. Williams

**Affiliations:** ^1^ Environmental Medicine/Radiation Oncology University of Rochester Medical Center Rochester New York USA

Dear Editor

We appreciate the points made by Dr. DiCarlo‐Cohen and thank her for the clarifications. Indeed, we fully acknowledge the significant contributions that she and the Radiation and Nuclear Countermeasures Program (RNCP) have provided to the field of radiation biology over the past two decades. However, with respect to the first point, the inflation rate since 2004 has been roughly 2.50% per year between 2004 and the present, resulting in a cumulative price increase of nearly 60%. Thus, to simply maintain funding at the initial level would require a current allocation of $73.5 million per year, and not the 30% decrease in real‐time costs. Of note, and as is pointed out, the appropriations in this area are earmarked for high dose, and not low dose, radiation, the chief focus of our published series of reports. With respect to the misrepresentation of the role of BARDA in the CMCR program, we sincerely apologize. Finally, we are aware of, and grateful for, the RNCP's consistent support of the development of educational materials, including the web‐based publication of the CMCR Radiobiology and Methods Public Textbook. However, important as educational materials are, they are no substitute for radiation biologists, who are needed to teach, mentor, and inspire students to pursue careers in this discipline. Furthermore, having expert scientists as mentors in graduate and postdoctoral programs provides the additional possibility of training in the laboratory setting, helping to cement the expertise of the next generation of scientists.

We thank Dr. DiCarlo‐Cohen and RNCP for their support and contributions to the field of radiation biology and apologize for any perceived inaccuracies. However, the authors believe that the fundamental conclusions that the workforce in radiation biology is at a critical juncture in its existence remain true. Without a concerted effort to both stabilize and expand the radiation biology workforce, preserving its fundamental knowledge base whilst providing opportunities to progress, the discipline will likely face the professional equivalence of extinction in the not‐too‐distant future.

Jacqueline P. Williams, PhD, FASTRO

I thank the following colleagues for their assistance with the radiation biology workforce publication and their support and suggestions in the preparation of this response: Mitchell Anscher (Virginia Commonwealth University School of Medicine), Amy Kronenberg (Lawrence Berkeley National Laboratory), Theodore Lawrence (University of Michigan—University Hospital), Brian Marples (University of Rochester Medical Center), Wayne Newhauser (Louisiana State University), Marcelo Vazquez (Loma Linda University), Jeffrey S. Willey (Wake Forest School of Medicine), Gayle E. Woloschak (Northwestern University), and Rosemary Wong (ret.—National Cancer Institute).

